# Supplementary material for: Cadmium-induced impairment in growth, photosynthetic apparatus and redox regulation in green amaranth (Amaranthus viridis L.) plant attenuated by salicylic acid and methyl jasmonate
Source: PLoS One. 2025 Aug 1;20(8):e0329373. doi: 10.1371/journal.pone.0329373 (PMC12316300; doi:10.1371/journal.pone.0329373)
Supplement: Attachment — Submitted filename: Response to Reviewers.docx [file pone.0329373.s002.docx]

**Response to Reviewers’ Comments for the Manuscript (PONE-D-25-15737)**

**Response to comments of Reviewer #1**

**Reviewer Comments:**

- The work is interesting and may have practical implications.
- I would suggest authors improve the conclusions and add strong suggestions for other studies:

**Authors’ response:**

- We would like to express our sincere thankfulness for your appreciation and efforts spent reviewing this paper, which will undoubtedly improve the manuscript's exposure and quality. We have carefully considered your suggestions and corrected them accordingly.
- The conclusion is improved and suggestions for future studies are now added in both the discussion and conclusion parts (Lines 700–708, 720–727).

**Reviewer Comments:**

- The sentence starting with "Keywords;" should end with a period instead of a semicolon.
- There are abbreviations which need elaboration
- There is need to add hypothesis in the introduction.
- Please be consistent for general name of plants or scientific names throughout the article
- Scientific names of plant species must in italics. Check it throughout the manuscript.
- Sometime methods have not been fully explained and sometime proper references are missing in this section

**Authors’ response:**

- The sentence starting with Keywords is now corrected
- All the abbreviations in the entire manuscript are checked and elaborated.
- The hypothesis of the research is now included in the introduction (Lines 107–110)
- The entire manuscript is updated and consistent with the common names of the plants
- The scientific names of plants are corrected with italic forms including the reference section
- The methodology has been thoroughly revised with more explanation of measured traits and missing and relevant references are added.

**Reviewer Comments:**

- Ensure consistency in terminology and notation throughout the results section.
- Properties of soil especially initial soil selected properties can be added in one or two sentences in the article.
- Sometime sentences are too short in length. Check this thoroughly in the whole article.
- Treatment application method is not clear
- Make sure that each figure has a clear and concise caption or legend. This should briefly explain the key findings or context of the figure for readers who may not closely examine the results section.
- There are many subheadings in this section and most of the subheadings are lengthy
- Sentence structure is very poor in this section in results section

**Authors’ response:**

- The results section is thoroughly and carefully checked. Terminology and notations are now consistent throughout the results section. Please have a look at the results section.
- We have not used soil as a medium of plant growth and therefore did not mention soil properties.
- Short sentences are elaborated and rephrased in the entire manuscript
- The treatment application method is updated and clarified (Lines 142–149)
- The figure captions are revised and made concise and informative. Missing legends are added.
- Subheadings in the results sections are now amended with shortened form
- We have carefully considered the sentence structure in the result section and revised it accordingly

**Reviewer Comments:**

- In some instances, it would be beneficial to explain how the cited studies are relevant to the current study in discussion section.
- Discussion should be rewritten to explain mechanistic explanation rather than superficially covering the topic
- Conclusion is too short.
- It's valuable to include a paragraph on the potential applications of the findings and suggest directions for future research. For example, how could this knowledge be applied in agriculture, and what specific experiments or investigations might be warranted?
- Figure looks properly prepared except missing legends in few figures
- Kindly add following references to make MS more catchy
  https://link.springer.com/article/10.1007/s12210-018-0702-y
  https://doi.org/10.1007/978-3-030-27165-7_7
  https://doi.org/10.1007/s00344-018-9854-3
  https://doi.org/10.1007/s12298-019-00715-y
  https://doi.org/10.3389/fpls.2022.895427

**Authors’ response:**

- The discussion part is modified with an emphasis on relevancy/connection between cited studies and the current study.
- The discussion is revised with more mechanistic explanation where necessary (Lines 532–660, 576–580, 630–647, 656–683, 700–708).
- The conclusion is revised and elaborated with more useful findings
- The potential applications of the findings and the direction of future research are now added as a new paragraph in the discussion section (Lines 700–708) and mentioned in the conclusion (Lines 720–723).
- Missing legends in the figures are incorporated.
- The suggested relevant citations are included in the introduction and discussion part (Reference numbers – 18, 20, 31, 39, 85)

Thanks again for your valuable time and please check the revised manuscript.

**Response to comments of Reviewer #2**

**Reviewer Comments:**

- Thanks to the authors for this manuscript. This is a well-written and informative piece of work. The introduction clearly establishes the context, problem, and purpose of the study. The methods sections is well-detailed and crafted, although some sentences are a bit long and could be slightly shortened for better readability (I recognize this is subjective). The discussion is well done and coherent with the results, although some phrases might be a bit speculative and I would either expand them with more information on the underlying mechanisms that support the considerations, or tone them down.
- The manuscript could be published as it is, since it is of good quality, but I have some suggestions for minor improvements if the authors accept them:

**Authors’ response:**

- We wish to express our sincere gratitude for taking the time to review this manuscript. We appreciate your valuable feedback which will certainly improve the quality of the manuscript. We have respectfully considered your valuable suggestions and revised the manuscript accordingly – in particular the discussion part.

**Reviewer Comments:**

- "Increased population growth in Bangladesh forces rapid industrialization and imprudent use of agrochemicals (heavy metal-containing pesticides and fertilizers)".
  I agree that the "imprudent use" aspect is important to emphasize, but it's not inherent to population growth. It might be more accurate to say that the increased demand for food production drives agrochemical use, which might be or not abused depending on various aspects (for example, local laws).
- "Heavy metals incorporate into the food chain through plant uptake via soil-root-crop or water-root-crop pathways triggering serious health hazards for living organisms (4,5).
  I would slightly rephrase this saying that heavy metals *can* incorporate (depending on the plant species, the metal, and soil conditions) and *might* trigger health hazards, also depending on amounts.

**Authors’ response:**

- We agree with your points. The sentence is now revised according to your viewpoints (Lines 42–46).
- We have rephrased the mentioned sentence according to your suggestion (Lines 47–50)

**Reviewer Comments:**

- "A well-known cultivated variety “Gheekanchan” from Lal Teer Seed Ltd. was chosen for this experiment."
  Can you provide a brief justification for choosing this specific variety? e.g. is it known for its Cd accumulation capability, its popularity among local farmers, or some other relevant characteristic? Even if it is just because it was simple to get and manipulate.
  Also, I suggest to invert the order of words as "For this experiment, a well-known etc." (stylistic choice)
- "amaranth seeds were disinfected with Vitavax-200"
  Can you provide a short description of this product? In particular, describing why you chose this one in particular, with which some scholars might be unfamiliar.
- "(95%) solution was purchased from Sigma-Aldrich, Germany and 11.8 µL was used to prepare a 1mM stock solution for 50 mL volume. The SA (Sigma Aldrich, Germany) stock solution (1 mM L-1) was made by dissolving 138.2 mg in 2 mL methanol and then adding distilled water as needed"
  Could you verify thins? Is 11.8 µL of 95% MeJA in 50 mL truly 1 mM? Or should it be a different volume? Is 138.2 mg/L of SA correct, given the molecular weight (molar mass)?

**Authors’ response:**

- Thanks for the valuable point. The availability of green amaranth cultivars is limited in Bangladesh and this variety was chosen among others due to its suitability for round-the-year production, easy availability, fast and vigorous growth, attractive green leaves, softness and deliciousness. We have now added the justification for choosing this variety (Lines 119–124). Also, the sentence is reformed according to your suggestion
- A short description of the product Vitavax-200 is presented and justification for selecting this product is mentioned (Lines 124–128)
- The calculation and formulation of MeJA and SA solution preparation are correct. MeJa was purchased from Sigma Aldrich as a 95% solution. Please have a look at the following figure for the MeJA calculation in detail. The molecular mass of SA is 132.12 g/mol which means, for 1 M solution, we need to add 132.12 g to one liter of deionized water. Therefore, we added 132.12 mg to one liter which is equivalent to 1 mM with a final volume of one liter.

**Reviewer Comments:**

- "Fourteen seedlings were supported by a perforated cork sheet in each tank and regarded as a single replicate. "
  I suggest to expand this for researchers trying to replicate the study or use it as a base for their projects, so that they will be able to handle with flexibility the given info. Only fourteen seedlings in total might be considered a bit little for accurate statistics, while putting them in just one sheet might be a bit crowded (depending on the size of the sheet). Could you add some text specifying why the outcome should not be compromised and is well reliable?
- "Cd stress was initiated in hydroponic conditions (21 d old seedlings) by introducing 10 µM CdCl2·H2O in the nutrient solution in all treatments except control and the duration of Cd exposure was three weeks."
  I suggest to specify why you chose this specific cadmium concentration and why exactly 21 days seedlings, even if you just found it to be a practical and workable setup. You later say "The Cd concentration was chosen based on a prior trial.", is it published data that you could cite, or just internal screening? Could you give further info? This can be useful for other researchers wanting to replicate the experiment who might discuss whether to follow by the book the protocol or slightly alter it depending on the situation.
- "The composition was kept in the dark to initiate the reaction and then the absorbance was recorded at 390 nm wavelength."
  You mean, immediately put in the dark and then you measured absorbance?

**Authors’ response:**

- We used a single tank as one replicate, each containing 14 seedlings. A total of three such tanks (replicates) were maintained per treatment, resulting in 42 plants per treatment group. This has now been clearly stated and elaborated in the revised methodology section (Lines 146–149). While placing 14 seedlings in a single sheet did create some crowding—especially for control plants toward the end of the experiment—we managed the setup effectively over the 42-day growth period. Nonetheless, we acknowledge this limitation and will take it into consideration in future experiments.
- Prior to selecting cadmium (Cd) treatment levels, we conducted an internal screening using green amaranth to determine appropriate concentrations. This information has now been added to the methodology section (Lines 154–158). For a practical and workable setup, seedlings were first established over a 21-day period, followed by 21 days of Cd exposure. Based on findings from our recent study and supporting literature, we hypothesized that 21 days would be sufficient to elicit measurable responses in leafy vegetables to Cd stress. Additionally, this duration was selected to align with the typical harvest period (40–45 days) for green amaranth. Further clarification on this experimental design is now included in the methodology section (Lines 158–163).
- The extract was kept in the dark for one hour before absorbance readings were taken. We have corrected the relevant sentence in the revised manuscript (Line 220)

**Reviewer Comments:**

- "MeJA and SA supplementation might counteract growth inhibition by facilitating cell division and enlargement and avoiding cell damage."
  Can you describe the known mechanisms of action for this and similar passages? This can be useful for some latter phrases which hypothesize possible consequences from it.
- "Under Cd stress, MeJa may help in controlling other growth-promoting hormones such as gibberellin and ethylene enabling plant growth and development (63)."
  I suggest to replace "enabling" with "promoting".
- "The supplemented MeJA and SA may have increased their endogenous levels that would restore cell permeability modulating nutrient uptake, gene expression and redox balance under Cd stress (65)"
  This is a bit speculative, since you didn't measure that. I would rephrase in more cautious words, and give a possible mechanism supporting this idea.
- "In addition, Cd stress may induce perturbation" [...] "The degraded photosynthetic pigments along with the impairment of the light-harvesting complexes and both PSI and PSII under Cd stress may have resulted in a declined rate of photosynthesis (A)"
  As above, these phrase could be a bit speculative. I recognize that they can be legitimate, so take my suggestion as a way to make your conclusions stronger and clearer for the reader.

**Authors’ response:**

- This part of the discussion is now revised with known mechanisms of MeJA and SA in relation to cell division, enlargement and growth stimulation under Cd stress with latest and relevant references (Lines 532–545)
- The sentence is revised according to your suggestion
- We are in full agreement with your point of view. The role of endogenous SA and MeJA in modulating plant growth and physiology is now discussed with supporting references. We have extensively modified this part of the discussion (Lines 545–560 ).
- Similar to the above comment, considering your viewpoints, we have revised this specific part accordingly (Lines 576–582). In addition, we thoroughly revised the discussion section in a mechanistic approach incorporating more recent and relevant citations. Thanks again for your valuable comments and time for reviewing the manuscript.
